# Oxidative Status Imbalance in Patients with Metabolic Syndrome: Role of the Myeloperoxidase/Hydrogen Peroxide Axis

**DOI:** 10.1155/2014/898501

**Published:** 2014-10-15

**Authors:** Lucas José Sá da Fonseca, Valéria Nunes-Souza, Glaucevane da Silva Guedes, Glauber Schettino-Silva, Marco Antônio Mota-Gomes, Luíza Antas Rabelo

**Affiliations:** ^1^Laboratório de Reatividade Cardiovascular, Setor de Fisiologia e Farmacologia, Instituto de Ciências Biológicas e da Saúde (ICBS), Universidade Federal de Alagoas (UFAL), Avenida Lourival Melo Mota, s/n, Cidade Universitária, 57072-900 Maceió, AL, Brazil; ^2^Instituto Nacional de Ciência e Tecnologia em NanoBiofarmacêutica (N-BIOFAR), Avenida Antônio Carlos, s/n, Pampulha, Belo Horizonte, MG, Brazil; ^3^Max-Delbrück-Center for Molecular Medicine, Robert-Rössle-Strße 10, 13125 Berlin, Germany; ^4^Faculdade de Nutrição (FANUT), Universidade Federal de Alagoas (UFAL), Avenida Lourival Melo Mota, s/n, Cidade Universitária, 57072-900 Maceió, AL, Brazil; ^5^Centro de Pesquisas Clínicas do Hospital do Coração de Alagoas (HCOR-AL), Avenida Ariosvaldo Pereira Cintra 152, Gruta de Lourdes, 57052-580 Maceió, AL, Brazil

## Abstract

The present study evaluated the cardiometabolic and redox balance profiles in patients with Metabolic Syndrome compared to apparently healthy individuals, and the participation of the myeloperoxidase/hydrogen peroxide axis in systemic lipid peroxidation. Twenty-four patients with Metabolic Syndrome and eighteen controls underwent a full clinical assessment. Venous blood samples were collected for general biochemical dosages, as well as for the oxidative stress analyses (superoxide dismutase, catalase, and arginase activities; and lipid peroxidation, myeloperoxidase activity, nitrite, and hydrogen peroxide concentrations in plasma). Arterial stiffness was assessed by radial artery applanation tonometry. Plasma lipid peroxidation, erythrocyte superoxide dismutase activity, myeloperoxidase activity, and hydrogen peroxide concentrations were shown to be increased in Metabolic Syndrome patients, without significant differences for the other enzymes, plasma nitrite concentrations, and arterial stiffness. Linear regression analysis revealed a positive and significant correlation between lipid peroxidation and myeloperoxidase and also between this enzyme and hydrogen peroxide. In contrast, such correlation was not observed between lipid peroxidation and hydrogen peroxide. In summary, Metabolic Syndrome patients exhibited evident systemic redox imbalance compared to controls, with the possible participation of the myeloperoxidase/hydrogen peroxide axis as a contributor in lipid peroxidation.

## 1. Introduction

Cardiovascular diseases represent the leading cause of death worldwide and may result from the association of different cardiometabolic risk factors [1]. When such factors simultaneously cluster in the same individual, they contribute to the establishment of Metabolic Syndrome (MetS), a condition characterized by the combination of increased blood pressure and glycemic levels, dyslipidemia, and abdominal obesity [2–4], which directly increases the risk of cardiovascular disease in its carriers [1, 2]. Indeed, the MetS participates in various pathological metabolic processes, with possible negative outcomes on the general biochemical profile [5], redox balance [4–6], and arterial function [1, 7].

When considering the MetS diagnostic parameters, a huge body of evidence points to their relation with the oxidative stress [4, 5, 8]. The latter, also known as redox imbalance, is characterized by a condition in which the excess of reactive oxygen and nitrogen species (RONS) compromises or even surpasses the action of endogenous antioxidant systems, either for increase in prooxidants, such as RONS, or for decreased antioxidant defense [9]. The oxidative stress implies pleiotropic toxic effects on cellular metabolism [4, 10], with potential damage to different organic systems [5, 11], especially in the vasculature [10, 12]. Concerning the association between oxidative stress and the MetS, literature reports are not consensual when it comes to the pattern of redox balance in patients presenting with MetS [6, 8, 13].

Myeloperoxidase (MPO), a heme peroxidase abundantly expressed in leukocytes, is a central enzyme in innate host defense [14, 15]. Primarily stored in cytoplasmic granules [16], MPO may be released to the extracellular compartment after phagocyte activation [17, 18]. Using hydrogen peroxide (H_2_O_2_) as a cosubstrate, MPO participates in the formation of different oxidants, among which are hypohalous acids [16]. Despite its beneficial effects related to leukocyte-mediated protection against pathogens, its excessive activity may imply tissue damage through oxidant production [15], being involved in chronic inflammatory conditions [14], among which are atherosclerosis and coronary artery disease [19], and also promoting endothelial dysfunction [20]. In this respect, however, even though several lines of evidence point to the association between the parameters related to the MetS and oxidative stress [12, 21, 22], the in-depth mechanisms involved in the pathophysiology of the MetS, particularly those related to its components, oxidative stress, and arterial function, still remain poorly understood. Based upon this scenario, the present work aims to assess the oxidative stress profile in patients with MetS and their arterial status, and also to evaluate a possible pathway through which oxidative stress markers may contribute to systemic lipid peroxidation.

## 2. Materials and Methods

### 2.1. Subjects

**Figure 1:**
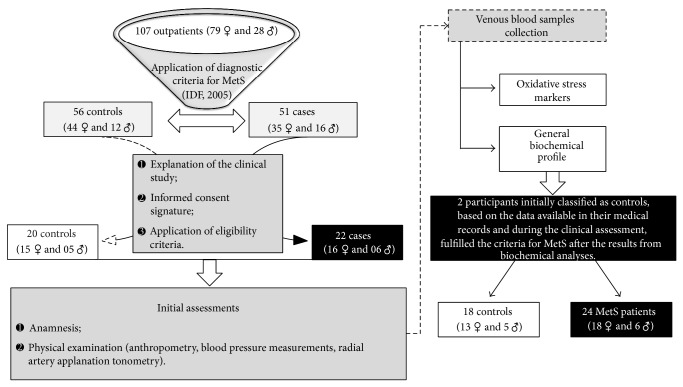
Study flow-chart. MetS: Metabolic Syndrome.

For this case-control study, a total of twenty-four patients with MetS and eighteen apparently healthy subjects were nonprobabilistically selected from the ambulatory of Endocrinology at the Teaching Hospital of the Federal University of Alagoas and from the adjacent community, respectively. Patients were paired for age, sex, tobacco use, dietary habits, and socioeconomic status. The steps from selection until the clinical and biochemical assessments are presented in Figure 1. The study protocol was approved by the Ethical Committee of the Federal University of Alagoas (Protocol number 010501/2009-91) and was in accordance with the principles outlined in the Declaration of Helsinki. All subjects gave written informed consent before carrying out the procedures.

The diagnosis of MetS was made based on the criteria defined by the International Diabetes Federation [23]. For both groups, exclusion criteria were age <30 or >65 years, patients taking antioxidant supplements, individuals presenting abnormalities which precluded the arterial assessment using radial artery applanation tonometry (e.g., patients with known arrhythmia, using pacemakers and those in which the left radial artery palpation could not be properly performed), pregnant or lactating women, patients undergoing hormone replacement therapy, abuse of alcohol, overt cerebrovascular, kidney or liver diseases, malignancies, as well as those who refused to participate in the study.

### 2.2. Clinical Assessment and Anthropometry

Initially, patients underwent a full anamnesis and physical examination. For brachial blood pressure measurements, a validated oscillometric device was used (Microlife, Widnau, Switzerland), with the cuff properly adapted to arm circumference. Three consecutive measures were taken, separated by one-minute interval each. The last two measures were averaged in order to obtain the mean for SBP and DBP. Heart rate (HR) was obtained simultaneously, with the last two measures averaged. Mean arterial pressure (MAP) was calculated according to the formula: MAP = (SBP + 2DBP)*·*3^−1^. Pulse pressure (PP) was obtained by subtraction between SBP and DBP.

Body weight was assessed using a precision digital scale (Filizola, São Paulo, Brazil) to the nearest 0.1 kg, with the individuals in standing position, barefoot and wearing light clothes. Height was measured at the highest head point to the nearest 0.5 cm, with a stadiometer coupled to the scale and the head in anatomical position. Body mass index (BMI) was defined as the ratio between the body weight and the square of the height, expressed in kg*·*m^−2^.

Waist circumference (WC, to the nearest 0.5 cm) was measured using a heavy-duty inelastic fiberglass tape (Cardiomed, Curitiba, Brazil) placed horizontally and tension free immediately over the skin at the midpoint between the last rib and the iliac crest, with measurements taken at the end of expiration. Neck circumference (NC) was assessed with the head in anatomical position, and the fiberglass tape placed right above the superior margin of the laryngeal prominence, parallel to the horizontal plane. Hip circumference (cm) was measured taking as reference the largest circumference on the hip anatomy, and waist-to-hip ratio (WHR) was then calculated. All anthropometric measurements were performed in the morning by an only physician previously trained.

### 2.3. Noninvasive Assessment of the Arterial Function

For noninvasively assessing the arterial function, the method of radial artery applanation tonometry was used, as described elsewhere [24]. All results were expressed as the average of the three measures captured by the sensor. The procedures were performed by a previously trained investigator broadly familiarized with the method.

### 2.4. Blood Samples Collection and General Biochemical Profile

For the biochemical analysis, patients underwent peripheral venous blood collection from an antecubital vein after a 12-hour overnight fasting. Immediately after, samples destined to the determination of general biochemical profile were processed according to standard laboratory techniques. Analytes for assessing the redox state were rapidly put in ice bath (4°C), being centrifuged at 1600 g for 10 minutes (Fanem, São Paulo, Brazil) to separate plasma from blood cellular elements. Next, plasma and erythrocyte samples were aliquoted and stored at −80°C until analysis.

### 2.5. Nonesterified Fatty Acids (NEFA) in Plasma

The NEFA quantification in plasma samples was assayed using a commercial kit (Wako Chemicals GmbH, Neuss, Germany), according to the manufacturer's protocol, with adaptations for microplate (Thermo Fisher Scientific, Vantaa, Finland). The results were expressed in mmol*·*L^−1^.

### 2.6. Fasting Insulin in Plasma

For quantitative insulin detection in plasma, a commercial ELISA kit was used (Millipore, Missouri, USA), following the manufacturer's instructions. A standard curve was used to determine insulin concentrations, with the results expressed in mU*·*L^−1^.

### 2.7. Assessment of Insulin Resistance

The degree of insulin resistance was estimated using the mathematical model HOMA-IR (Homeostasis Model Assessment − Insulin Resistance) index and calculated as follows: HOMA-IR = [fasting insulin (*μ*U*·*L^−1^) × fasting glucose (mmol*·*L^−1^)]/22.5  [25]. Values were expressed in *μ*U*·*L^−1^/mmol*·*L^−1^. High HOMA-IR values indicate a state of insulin resistance, while low HOMA-IR values are associated with better insulin sensitivity.

### 2.8. Estimation of the Glomerular Filtration Rate

Estimated glomerular filtration rate (eGFR) was calculated using the simplified MDRD (Modification of Diet in Renal Disease) formula, as follows: 186 × plasma creatinine^−1.154^  × age^−0.203^  × 1.212. For women, results were further multiplied by the constant 0.742 [26].

### 2.9. Lipid Peroxidation in Plasma

The lipid peroxidation in plasma was quantified based on the protocol described by Ohkawa et al. [27], with slight adaptations, for determining the Thiobarbituric Acid Reactive Substances (TBARS), among which malondialdehyde (MDA) figures as the most representative one. Absorbance was read in a microplate reader (Thermo Fisher Scientific, Vantaa, Finland), at wavelengths of 532 nm and 600 nm. The dosages were performed in duplicate and TBARS values were normalized by total protein concentration in plasma [28], and expressed as *μ*M*·*[Protein] mg*·*mL^−1^.

### 2.10. Erythrocyte Lysates and Measurement of Hemoglobin Concentrations

After thawing in ice bath (4°C), 250 *μ*L of erythrocyte samples were taken for hemolysis, being aliquoted and stored at −80°C, until the redox analyses were performed. Hemoglobin (Hb) concentrations in erythrocyte lysates were measured using a commercial kit (Labtest, Belo Horizonte, Brazil), according to the manufacturer's protocol.

### 2.11. Total Superoxide Dismutase (SOD) Activity in Erythrocytes and in Plasma

Superoxide dismutase (SOD) activity was determined in erythrocyte lysates and in plasma, being read in microplate (Nunc, Roskilde, Denmark), using a commercial kit (Fluka, Sigma-Aldrich, St. Louis, USA), according to the manufacturer's protocol, at a wavelength of 450 nm (Thermo Fisher Scientific, Vantaa, Finland). Values were normalized by Hb concentrations and expressed in IU*·*mg Hb^−1^ in erythrocytes and by the total protein concentration [28] in plasma, with results expressed in IU*·*mg Protein^−1^.

### 2.12. Catalase Activity in Erythrocytes and in Plasma

Catalase activity was measured in erythrocyte samples and in plasma in microplates (Nunc, Roskilde, Denmark), according to the protocol described by Xu et al. [29] and expressed in IU*·*mg Hb^−1^ in erythrocytes. For catalase activity in plasma, samples were directly plated in the wells, without dilution, before the reagent addition. Values were normalized by total protein concentration [28] and the enzyme activity, expressed as IU*·*mg Protein^−1^.

### 2.13. Hydrogen Peroxide (H_2_O_2_) Concentrations in Plasma

The quantification of plasma H_2_O_2_ levels was performed by fluorescence (Tecan 200 Infinite, Männedorf, Switzerland), with a commercial kit (Ultra Amplex Red Hydrogen Peroxide/Peroxidase Assay kit, Invitrogen, Paisley, UK), according to the manufacturer's instructions. In the presence of peroxidase (horseredish peroxidase, HRP), the Amplex Red reagent stoichiometrically reacts with H_2_O_2_ to form a red-fluorescent oxidation product, resorufin. A standard curve of H_2_O_2_ was prepared, with concentrations ranging from 0 to 10 *μ*M. Next, 50 *μ*L from the curve points or from the samples were plated in duplicate, with the addition of 50 *μ*L of the reagent/HRP solution to start the reaction. Finally, the black microplates (Nunclon Surface, Thermo Fisher Scientific, Vantaa, Finland) were incubated at room temperature for 120 minutes, protected from light and read at wavelengths of 530 and 590 nm, respectively, related to excitation and emission.

### 2.14. Myeloperoxidase (MPO) Activity in Plasma

Similarly to the determination of H_2_O_2_ levels, MPO activity in plasma samples was performed using the Ultra Amplex Red Hydrogen Peroxide/Peroxidase Assay kit (Invitrogen, Paisley, UK), according to the manufacturer's instructions. A standard curve was prepared, with MPO concentrations ranging from 0.0312 to 1.0 UI*·*mL^−1^ (Sigma, St. Louis, USA). Then, 50 *μ*L of the curve points or from the samples were plated, with the addition of the Amplex Red/H_2_O_2_ working solution to start the reaction. Next, samples were incubated at room temperature for 150 minutes, protected from light. Finally, fluorescence was measured in a spectrofluorometer (Tecan 200 Infinite, Männedorf, Switzerland), using black microplates (Nunclon Surface, Thermo Fisher Scientific, Vantaa, Finland) at the wavelengths of 530 and 590 nm for excitation and emission, respectively.

### 2.15. Plasma Levels of Nitrite

The quantification of plasma nitrite concentrations was performed based on the protocol described by Misko et al. [30], with adaptations for microplates. This fluorimetric assay is based on the reaction between nitrite and the compound 2,3-diaminonaphthalene (DAN), originating 2,3-diaminonaphthotriazole. Initially, plasma samples were filtered using a 10 dKa molecular weight filter (Millipore, Missouri, USA). Then, using black 96-well microplates (Nunclon Surface, Thermo Fisher Scientific, Vantaa, Finland), to 50 *μ*L of each sample (in duplicate) were added 100 *μ*L of deionized water. Next, 10 *μ*L of DAN (0.05 mg*·*mL^−1^ in HCl 0.62 M) were added and mixed immediately, with DAN always protected from light. After incubation at 20°C for 10 minutes, the reaction was stopped by the addition of 5 *μ*L of NaOH (2.8 M). The compound formed was quantified in a spectrofluorometer (Tecan 200 Infinite, Männedorf, Switzerland), at 365 nm and 410 nm for excitation and emission, respectively. Nitrite concentrations were calculated based on a standard curve of nitrite.

### 2.16. Arginase Activity in Erythrocytes and in Plasma

Arginase activity was determined using a colorimetric method, as previously described [31], with adaptations for microassays. Briefly, erythrocyte lysates were dissolved in PBS 1 : 20 (v : v) and homogenized under cooling. Next, 50 *μ*L from the solution were incubated with 75 *μ*L of a Tris-HCl (50 mmol*·*L^−1^ plus 10 mmol MnCl_2_; pH 7.5) solution supplemented with manganese chloride (10 mmol*·*L^−1^) at 60°C in an incubator during 10 minutes. After this, the reaction was initiated by the addition of 50 *μ*L of the substrate L-arginine (100 mmol*·*L^−1^) and processed at 37°C for 1 hour. At the end of this step, 400 *μ*L of an acid solution were added in order to stop the reaction. The reagent *α*-isonitroso-propiophenone (25 *μ*L; 9% in EtOH) was then added to the mixture, following another reaction for 45 minutes at 100°C. Finally, samples were incubated in the dark at room temperature for 10 minutes before reading. Absorbance was measured at 540 nm in a microplate reader (Thermo Fisher Scientific, Vantaa, Finland). Data were normalized according to hemoglobin concentrations, and enzyme activity was expressed in mmol/min/mL*·*mg Hb^−1^. For assessing the arginase activity in plasma, samples were not diluted but directly plated in the wells, with the other procedures similar to those applied during the determination of activity in erythrocytes. Data were normalized according to the total protein concentrations [28], and enzyme activity was expressed in mmol/min/mL*·*mg Protein^−1^.

### 2.17. Statistics

Data were analyzed using GraphPad Prism, version 5.00 (San Diego, CA, USA), and normality was tested applying the Shapiro-Wilk test. For continuous variables with normal distribution, the Student's *t*-test was used. For variables not presenting Gaussian distribution, the nonparametric Mann-Whitney* U* test was applied. Continuous variables are presented as mean ± standard deviation (SD) and categorical variables, in percentage. Linear regression analysis was also performed and results were considered significant if *P* < 0.05.

## 3. Results

### 3.1. Sample Characterization

The sample characterization evidenced the predominance of female patients in both groups (Table 1). No significant differences were observed for age and height. As expected, for the MetS individuals, weight, BMI, WC, WHR, and NC were increased compared to the controls (Table 1). Regarding drug therapy, 18 (75%) among all MetS patients were under regular use of medications for treating hypertension, dysglycemia/type 2 diabetes mellitus, or dyslipidemia (Table 2). The participants in the control group were not under use of any pharmacological treatment.

### 3.2. A Hypertensive Pattern and a Marked Imbalance in Glucose Profile Were Found in MetS Patients, despite the Pharmacological Treatment

For the cardiovascular parameters, SBP, DBP, PP, and MAP were significantly higher in MetS patients than observed in the controls (Table 1). For HR, however, no difference was identified between groups (Table 1). Results concerning the glucose profile evidenced both higher fasting glucose and HbA1_C_ levels in MetS patients compared to the controls (Table 1). Fasting insulinemia levels and the degree of insulin resistance were also increased in MetS patients (Table 1).

### 3.3. Significant Dyslipidemia Accompanied the Dysglycemia in MetS Patients

MetS patients presented significant dyslipidemia, with higher levels for total cholesterol, triglycerides, and VLDL cholesterol compared to the controls (Table 1). Furthermore, MetS individuals presented higher values for triglycerides/HDL cholesterol and total cholesterol/HDL cholesterol ratios, without significant differences for plasma concentrations of HDL cholesterol, LDL cholesterol, and NEFA (Table 1).

### 3.4. MetS Patients Displayed Diminishment in Renal Function

When considering the parameters for renal assessment between groups, no significant difference for urea concentration in plasma was observed. Nevertheless, MetS patients showed decreased renal function compared to the controls, as observed by increased creatinine levels and lower eGFR (Table 1).

### 3.5. MetS Patients Presented Elevated Uricemia and Liver Enzymes, without Changes in Neutrophil Count and in High-Sensitivity C-Reactive Protein Levels

MetS individuals showed higher uric acid and AST and ALT concentrations in plasma, compared to the controls. Also, a reduction in the AST/ALT ratio was observed in the MetS group. However, for the hs-CRP levels, white blood cell count, and neutrophils, no differences between groups were observed (Table 1).

### 3.6. The Lack of Difference between Groups for Arterial Stiffness and Nitrite Concentrations in Plasma Was Accompanied by Similar Arginase Activity

**Figure 2:**
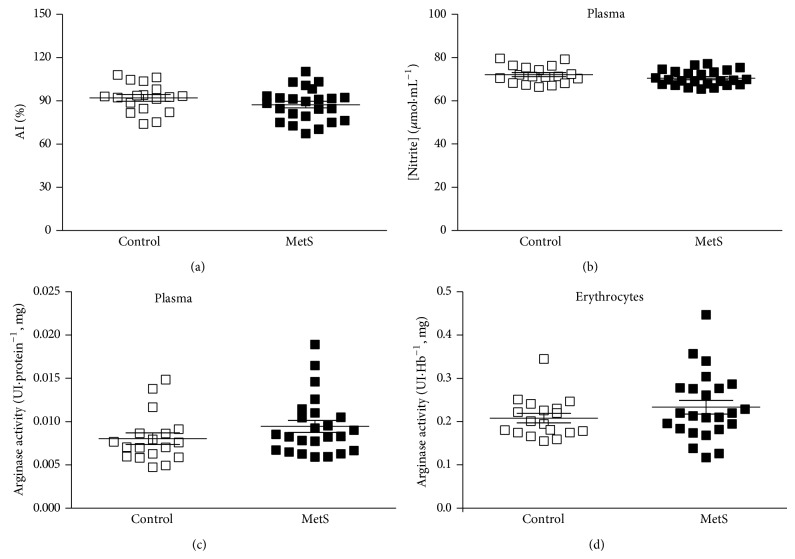
(a) Augmentation Index (AI), (b) nitrite concentrations, and ((c) and (d)) arginase activities in patients with Metabolic Syndrome (MetS; *n* = 24) and controls (*n* = 18). Student's *t*-test for AI and nitrite concentrations. Mann-Whitney *U* test for arginase activities.

Results for the arterial stiffness assessment did not show significant difference between groups for the AI (Figure 2(a)). The lack of difference between groups was also observed when quantifying the nitrite levels in plasma (Figure 2(b)). For the arginase activity, no differences were observed between groups, neither in erythrocytes (Figure 2(c)), nor in plasma (Figure 2(d)).

### 3.7. Increased Erythrocyte SOD Activity Was Not Accompanied by Changes in Catalase Activities

**Figure 3:**
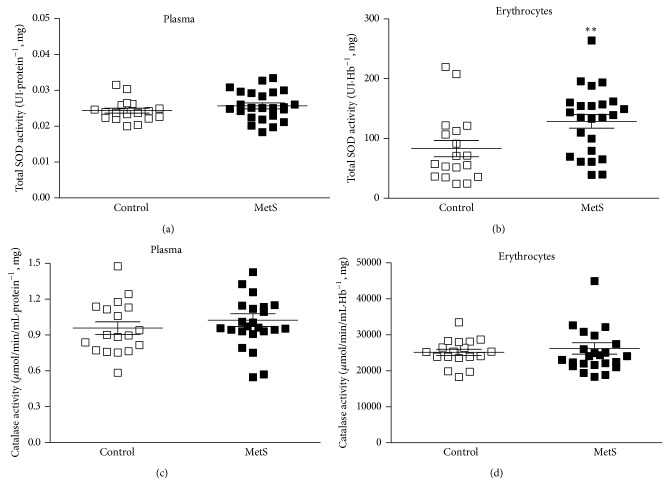
((a) and (b)) Superoxide dismutase (SOD) and ((c) and (d)) catalase activities in patients with Metabolic Syndrome (MetS; *n* = 24) and controls (*n* = 18). Student's *t*-test for SOD and catalase in plasma. Mann-Whitney *U* test for SOD and catalase in erythrocytes. ^**^ *P* < 0.01.

For total SOD activity in plasma, no significant difference was observed between groups (Figure 3(a)). For total SOD in erythrocytes, however, a significantly higher activity was found in MetS individuals (Figure 3(b)). Nevertheless, for the catalase activities, no significant differences between groups were identified (Figures 3(c) and 3(d)).

### 3.8. Augmented H_2_O_2_ Concentrations and MPO Activity May Contribute to Increased Lipid Peroxidation in Plasma

**Figure 4:**
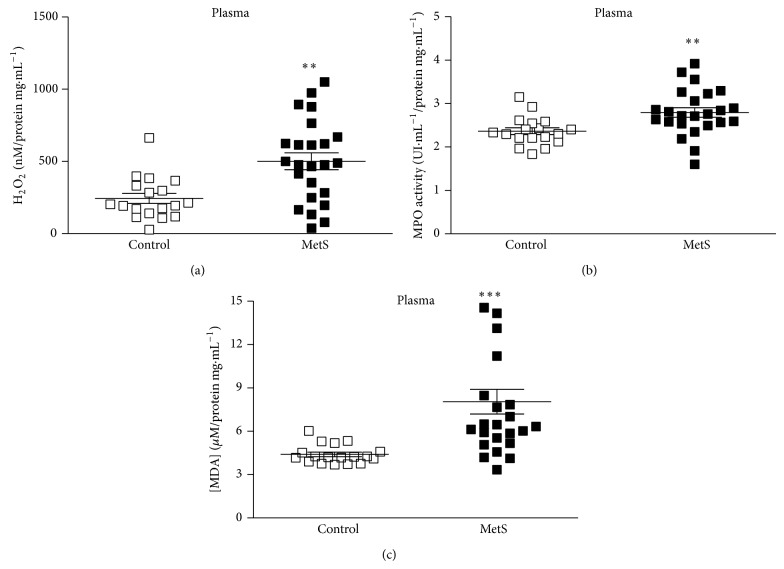
(a) Plasma hydrogen peroxide (H_2_O_2_) concentrations, (b) myeloperoxidase (MPO) activity, and (c) lipid peroxidation (MDA: malondialdehyde) in patients with Metabolic Syndrome (MetS; *n* = 24) and controls (*n* = 18). Student's *t*-test for H_2_O_2_ and MPO. Mann-Whitney *U* test for lipid peroxidation. ^**^ *P* < 0.01; ^***^ *P* < 0.0001.

**Figure 5:**
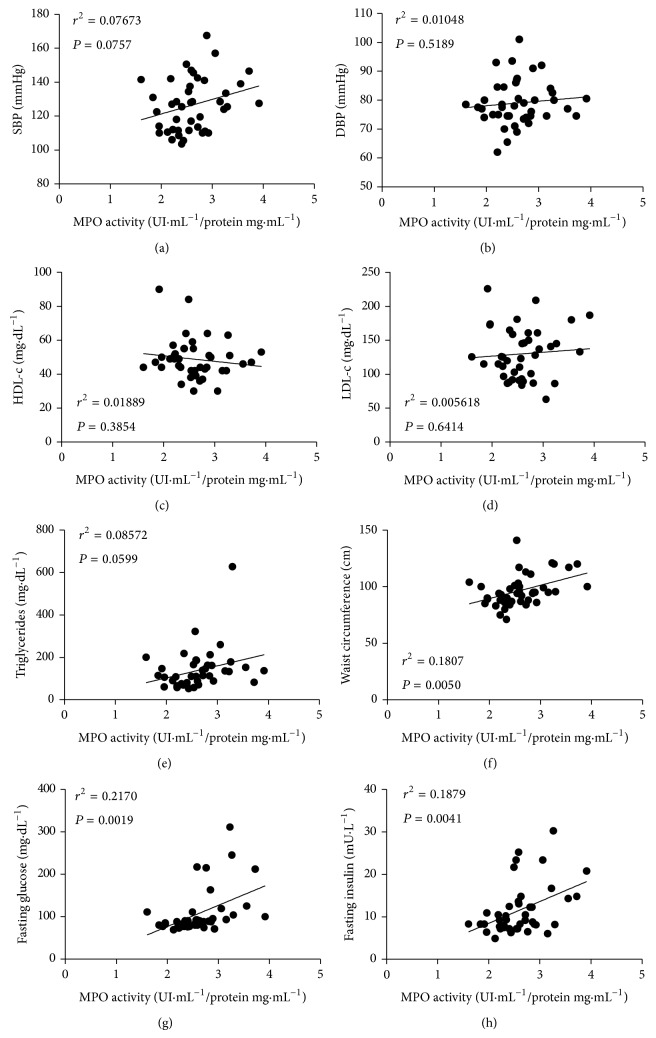
Linear regression analyses between myeloperoxidase (MPO) activity and (a) systolic blood pressure (SBP); (b) diastolic blood pressure; (c) HDL cholesterol (HDL-c) levels; (d) LDL cholesterol (LDL-c) levels; (e) triglycerides; (f) central obesity; (g) fasting glucose; and (h) fasting insulin. For LDL-c levels, *n* = 41 because in the MetS group one patient exhibited triglyceride levels greater than 400 mg*·*dL^−1^, thus impairing the determination of LDL-c levels. For the other parameters assessed, *n* = 42.

**Figure 6:**
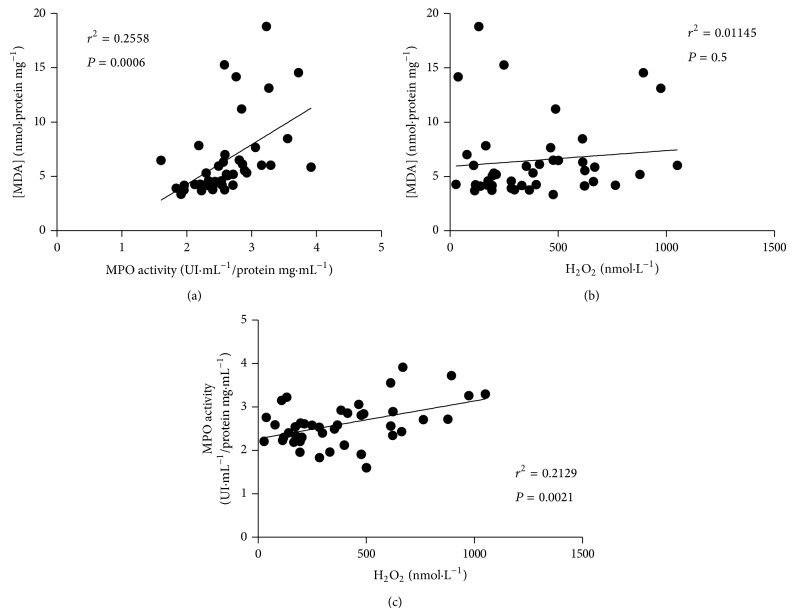
Linear regression analysis for the main oxidative parameters significantly altered between groups (*n* = 42). MPO: myeloperoxidase. H_2_O_2_: hydrogen peroxide. MDA: malondialdehyde.

The analysis of H_2_O_2_ in plasma evidenced higher concentrations in patients with MetS compared to the controls (Figure 4(a)). In the same direction of the observation for H_2_O_2_ concentrations, MPO activity in plasma was found to be increased in MetS individuals (Figure 4(b)). The assessment of lipid peroxidation in plasma showed a greater state of systemic redox imbalance in MetS patients, as observed by the increased MDA concentrations in such group compared to the controls (Figure 4(c)). Linear regression analyses revealed positive and significant correlations between two MetS components (WC and fasting glucose) and MPO (Figures 5(f) and 5(g), resp.) and also between fasting insulin and MPO (Figure 5(h)). For the other MetS components and LDL-c, no significant correlations were observed with MPO (Figures 5(a)–5(e)). For the oxidative stress markers MDA, MPO, and H_2_O_2_, significant correlations were found between MDA and MPO and also between MPO and H_2_O_2_ but not between MDA and H_2_O_2_ (Figure 6).

## 4. Discussion

The main findings of the present work point to the possible action of MPO on its cosubstrate, H_2_O_2_, amplifying the systemic lipoperoxidation, being unlikely the direct participation of such radical in this process, in the considered sample.

When assessing the oxidative status, a significant redox imbalance in the MetS group was observed compared to the controls, as identified by increased lipid peroxidation in the former. In this regard, a large body of evidence points to obesity as a critical determinant of systemic oxidative stress in humans [4, 32]. Thus, it is plausible to consider obesity, particularly that observed by increased WC, as a contributor for the augmented lipoperoxidation levels in MetS patients in this study. Indeed, we found a significant positive correlation between WC and MPO activity (Figure 5(f)). In line with these observations, Fujita et al. [33], in a case-control study, found increased levels of urinary 8-epi-prostaglandin F_2_ (a marker of systemic oxidative stress) in MetS carriers. Furthermore, such oxidative marker was shown to be strongly correlated with visceral obesity [33].

A significant dysglycemia, with increased insulin resistance, was another prominent feature of MetS patients in the present study. In this regard, our group showed that the increase in lipid peroxidation was positively correlated with fasting glucose and HbA1_C_ in diabetic patients [34]. These statements, together with the significant positive correlations between fasting glucose/fasting insulin and MPO activity (Figures 5(g) and 5(h)) in the current study, reinforce the probable participation of dysglycemia in the maintenance of the environment of redox imbalance in patients with MetS.

Our findings for lipid peroxidation are in line with those of Demircan et al. [13] and Armutcu et al. [8], as both groups observed, in case-control studies, increased plasma MDA levels in MetS individuals. In opposition to these findings, a case-control study with MetS patients by Sánchez-Rodríguez et al. [6] failed to show statistically significant differences between groups for lipoperoxidation. The apparent discrepancy between the aforementioned studies highlights the complexity with which the MetS presents itself in the clinical setting, so that it seems reasonable to consider other possible contributors for the state of lipid peroxidation identified, such as the accuracy of the methodologies used for estimating the degree of oxidative stress, age, the presence of comorbidities, and dietary habits [35]. We also found higher erythrocyte SOD activity in MetS patients. Indeed, SOD represents a first-line endogenous antioxidant defense, converting ^•^O_2_^−^ to O_2_ and H_2_O_2_ [36, 37]. Olusi, while studying the erythrocyte Cu-ZnSOD activity in obese individuals, observed reduced enzyme activity in these individuals, compared to counterparts without obesity. Viroonudomphol et al. [38], in turn, highlighted that SOD, by an adaptive response, may present augmented activity in states of increased lipoperoxidation, as a compensatory means to mitigate redox imbalance. Considering these observations, it is likely that the increased erythrocyte SOD activity found in the current study occurred as a compensatory response for opposing the increased lipid peroxidation.

Following the action of SOD on ^•^O_2_^−^, H_2_O_2_ may be converted to H_2_O and O_2_ under the actions of catalase or glutathione peroxidase (GPx) [10, 36]. Hence, in the present study, increased SOD activity was accompanied by augmented levels of H_2_O_2_, a substrate for catalase. However, no significant changes in catalase activities were observed. In view of the fact that GPx represents an antioxidant enzyme not assessed in our sample, it is not possible to exclude its participation in degrading H_2_O_2_.

MetS patients in the present study exhibited higher plasma MPO activity, compared to controls. This enzyme is already described as an important cardiovascular risk factor, capable of potentiating the oxidative effects of its cosubstrate, H_2_O_2_ [20].

In order to study the possible contribution of MPO activity and H_2_O_2_ concentrations in the determination of lipid peroxidation, linear regression analysis was performed, with the observation of a positive correlation between MPO activity and MDA plasma levels but not between H_2_O_2_ concentrations and MDA plasma levels (Figure 6). These data suggest the direct participation of MPO in increasing lipid peroxidation, with this fact not holding true for H_2_O_2_. Interestingly, the occurrence of a significant positive correlation between MPO activity and H_2_O_2_ (Figure 6) finally suggests that one of the pathways responsible for inducing lipid peroxidation seems to be strongly dependent on the action of MPO on its cosubstrate, H_2_O_2_, being unlikely the direct participation of such radical in this process. Considering the potential consumption of H_2_O_2_ by MPO, one could expect to observe an inverse correlation between such variables, as the increased MPO activity could respond for reduced H_2_O_2_ levels, but our data point to a direct correlation instead. This finding may be due to the fact that the augmentation in MPO activity and the H_2_O_2_ consumption are not occurring in a proportional manner, with the cosubstrate production surpassing its diminishment by MPO. The activity of this enzyme, albeit elevated in MetS patients, was not increased enough to determine reduced H_2_O_2_ levels, the latter proportionally higher as a consequence of the overt environment of redox imbalance observed.

After activation, by a process of degranulation, neutrophils release MPO, becoming depleted of this enzyme [18, 39]. Thus, despite the fact that no difference was observed between groups for neutrophil count (Table 1), it is reasonable to consider that elevated MPO activity in plasma samples of MetS patients may come from activated leukocytes. In addition, insulin resistance is responsible for augmenting the levels of proinflammatory mediators [40]. Furthermore, adipocytes, particularly in obese individuals, release inflammatory cytokines which compromise the insulin signaling [41]. Both of these factors (insulin resistance and central obesity) were found in MetS patients, but no differences were observed between groups for the marker of systemic inflammation hs-CRP. However, chronic inflammation is also associated with increased expression of MPO [14]. Literature highlights the association between proinflammatory mediators and the pathogenesis of the MetS, once dysregulation in inflammatory responses in the muscle and liver may be observed in the course of that syndrome [42]. Thus, the lack of difference for hs-CRP between groups and the significant increase in plasma MPO activity in the case group point to the potential use of such enzyme as an adjuvant marker for assessing the inflammatory state of patients with MetS.

Currently, it is already recognized that arterial stiffness is independently associated with the occurrence of cardiovascular events [43, 44]. Among the available parameters for noninvasively assessing the degree of arterial stiffness, the AI presents a strong correlation with the gold-standard (the Pulse Wave Velocity, PWV) for studying aortic stiffness [44], being an important marker of arterial stiffness [24].

Several studies have demonstrated the increased arterial stiffness in individuals with MetS [7, 45]. The AI and the PWV, albeit described as markers of vascular stiffness [44, 46], are not always altered in the same direction. Kovaite et al. [47] did not find significant difference for the AI obtained from the radial artery between patients with and without MetS. Nevertheless, the authors observed an association between MetS and the increase in PWV [47]. These data, together with those found in the present report for arterial stiffness, highlight the importance of considering the methodological approach used for determining the existence of significant differences between groups, without overlooking other possible interfering factors. In the current study, nine patients (37.49%) in the MetS group (Table 2) were taking angiotensin converting enzyme (ACE) inhibitors or angiotensin receptor antagonists, a fact that may have, at some degree, contributed to reduce the effects of the renin-angiotensin system in the vascular wall. In addition, statins may reverse abnormalities related to the arterial stiffness [43, 46]. Also, metformin is associated with reduction in macrovascular events in patients with diabetes [48]. Thus, the potential effects of such drugs must be considered when assessing the vascular stiffness in patients under their use.

More interestingly, the lack of difference between groups for the AI, a possible indirect means for assessing the endothelial function [49], was accompanied by similar concentrations of nitrite in plasma (also a marker of endothelial function) [12] in both groups.

The ^•^NO levels in individuals with MetS are rather conflicting in the literature. Simão et al., in a case-control study, identified lower concentrations of nitrite/nitrate in the MetS group, reflecting lower concentrations of ^•^NO [50]. In opposition to these observations, Ueyama et al. [51] showed that the concentrations of nitrite/nitrate increased when the number of the MetS diagnostic criteria was progressively augmented in humans. Such finding was defined as “unexpected,” and the authors suggested a possible compensatory pathway for increasing ^•^NO synthesis [51]. Taken together, such discrepancies may be related to the particularities of each considered sample, including the time of progression of the morbid process, and the presence and duration of pharmacological treatment. In this regard, in the present report, different drugs with potential to positively interfere on the endothelial function had been regularly used by MetS patients, such as ACE inhibitors, angiotensin receptor antagonists [52], metformin, and statins [53].

For arginase activity, no significant differences were observed between groups. This enzyme, predominantly expressed in the liver, kidneys, and erythrocytes, converts L-arginine to urea and ornithine [54]. Under conditions of increased arginase activity, a reduction in ^•^NO production may be observed, as such enzyme competes with eNOS for the same substrate, L-arginine [55]. In situations of metabolic injury, changes in arginase activity are already described, as was the case with the augmented basal serum arginase activity in diabetic patients compared to controls [54]. The lack of difference for arginase activity between groups in the current report may point to the unrepresentative participation of plasma and erythrocyte arginase in the cardiometabolic dysregulation observed in the MetS group. It is not possible, however, to rule out the participation of this enzyme in other tissue microenvironments not assessed in the present study.

Finally, the exact mechanisms that determine redox imbalance in MetS in humans have yet to be better dissected, but our findings give insight into the comprehension of an enzymatic pathway possibly involved in this process. Thus, we do believe that this observation may shed some light on the possibility of pharmacological strategies in order to mitigate the cardiometabolic derangements found in the course of MetS.

Some limitations deserve to be mentioned in this study. Firstly, the relatively small sample size does not ensure that the lack of significant difference for some parameters assessed really occurs in the general population, so that further studies are required to consider this possibility. Secondly, as an observational, case-control study, it is not possible to establish a causal relationship between the variables studied.

## 5. Perspectives

So far as we are aware, it is the first study pointing to the possible participation of MPO in amplifying the oxidative effects of H_2_O_2_ on systemic lipid peroxidation. Once lipoperoxidation was found to be dependent on the action of MPO, further studies are warranted to identify other possible contributing pathways in this process, but our data point to MPO/H_2_O_2_ as a potential therapeutic target in cardiometabolic diseases.

## Figures and Tables

**Table 1:** General, anthropometric, cardiovascular, and biochemical characteristics of participants included in the study.

	Control group (*n* = 18)		MetS group (*n* = 24)^a^		*P* values
General and anthropometric characteristics					
Gender	♂ (*n*/%) 5/27.77	♀ (*n*/%) 13/72.23	♂ (*n*/%) 6/25	♀ (*n*/%) 18/75	
Smoking	1/5.56		2/8.33		
Age (years)^b^	45.50 ± 7.45		50.17 ± 8.27		NS
Weight (kg)^c^	68.63 ± 11.04		79.86 ± 17.83∗		0.02
Height (m)^c^	1.60 ± 0.10		1.58 ± 0.10		NS
BMI (kg*·*m^−2^)^c^	26.69 ± 3.27		32.09 ± 7.10∗∗		0.0073
WC (cm)^b^	87.89 ± 7.65		103.30 ± 13.96∗∗∗		0.0001
WHR^b^	0.83 ± 0.06		0.90 ± 0.09∗∗		0.0042
NC (cm)^c^	34.92 ± 3.24		37.46 ± 5.99∗		0.03

Cardiovascular parameters					
SBP (mmHg)^c^	116.60 ± 9.18		134.00 ± 15.36∗∗∗		0.0007
DBP (mmHg)^b^	75.44 ± 5.92		81.75 ± 8.53∗∗		0.0078
MAP (mmHg)^b^	89.16 ± 5.94		99.16 ± 9.41∗∗∗		0.0003
PP (mmHg)^b^	41.14 ± 8.51		52.23 ± 12.24∗∗		0.0021
HR (bpm)^b^	72.58 ± 13.47		70.94 ± 12.68		NS

Glycemic profile, insulinemia, and insulin resistance					
Fasting glucose (mg*·*dL^−1^)^c^	80.11 ± 6.67		127.80 ± 64.10∗∗∗		<0.0001
HbA1c (%)^c^	4.99 ± 0.26		6.50 ± 1.66∗∗∗		<0.0001
Insulinemia (mU*·*L^−1^)^c^	8.95 ± 4.44		13.60 ± 6.21∗∗∗		<0.0001
HOMA-IR (*µ*U*·*mL^−1^)^c^	1.80 ± 1.06		4.58 ± 3.95∗∗∗		<0.0001

Lipid profile					
Total cholesterol (mg*·*dL^−1^)^b^	182.70 ± 29.83		225.60 ± 53.86^**^		0.0041
Triglycerides (mg*·*dL^−1^)^c^	81.39 ± 22.91		180.90 ± 110.30∗∗∗		<0.0001
HDL (mg*·*dL^−1^)^c^	49.17 ± 5.76		48.63 ± 15.08		NS
LDL (mg*·*dL^−1^)^b^	117.80 ± 28.78		139.70 ± 41.54		NS
VLDL (mg*·*dL^−1^)^c^	15.57 ± 4.47		36.18 ± 22.06∗∗∗		<0.0001
TG/HDL ratio^c^	1.69 ± 0.56		4.01 ± 2.48∗∗∗		<0.0001
TC/HDL ratio^c^	3.76 ± 0.72		4.81 ± 1.07∗∗		0.0008
NEFA (mmol*·*L^−1^)^b^	0.32 ± 0.10		0.36 ± 0.09		NS

Renal function parameters					
Urea (mg*·*dL^−1^)^b^	25.17 ± 7.13		29.58 ± 7.50		NS
Creatinine (mg*·*dL^−1^)^b^	0.77 ± 0.15		0.90 ± 0.18∗		0.0207
eGFR (mL/min/1.73 m^2^)^b^	119.00 ± 27.12		95.23 ± 21.92∗∗		0.0031

Others parameters					
WBC count (cells/mm^3^)^b,d^	6617 ± 1659		6400 ± 1744		NS
Neutrophils (cells/mm^3^)^b,d^	3934 ± 1553		3585 ± 1132		NS
Neutrophils (%)^c,d^	57.72 ± 11.09		55.71 ± 6.79		NS
Uric acid (mg*·*dL^−1^)^b^	3.31 ± 0.80		4.11 ± 1.14∗		0.0150
AST (U*·*mL^−1^)^c^	21.50 ± 8.05		32.63 ± 20.83∗		0.0127
ALT (U*·*mL^−1^)^c^	22.33 ± 9.77		46.88 ± 35.96∗∗∗		0.0006
AST/ALT ratio^b^	1.01 ± 0.29		0.78 ± 0.23∗∗		0.0063
hs-CRP (mg*·*L^−1^)^c^	2.77 ± 2.30		3.59 ± 3.94		NS

Values are expressed as mean ± standard deviation. ^a^For LDL-c levels in the MetS group, *n* = 23 because one patient exhibited triglyceride levels greater than 400 mg*·*dL^−1^, thus impairing the determination of LDL-c levels. For the other parameters assessed in the MetS group, *n* = 24. ^b^Student's *t* Test. ^c^Mann-Whitney *U* Test. ^d^Values obtained from peripheral venous blood samples. NS: nonsignificant. ALT: alanine aminotransferase; AST: aspartate aminotransferase; BMI: body mass index; DBP: diastolic blood pressure; eGFR: estimated glomerular filtration rate; HbA1c: glycated hemoglobin; HDL: high density lipoprotein cholesterol; HOMA-IR: homeostasis model assessment-insulin resistance; HR: heart rate; hs-CRP: high-sensitivity C-reactive protein; LDL: low density lipoprotein; MAP: mean arterial pressure; NC: neck circumference; NEFA: non-esterified fatty acids; PP: pulse pressure; SBP: systolic blood pressure; TC: total cholesterol; TG: triglycerides; VLDL: very low density lipoprotein; WBC: white blood cell; WC: waist circumference; WHR: waist-to-hip ratio. ^*^ *P* < 0.05; ^**^ *P* < 0.01; ^***^ *P* < 0.0001.

**Table 2:** Medications used by patients presenting with MetS.

Medications	(patients under use/total)	%
*β*-blockers	5/24	20.83
Diuretics	6/24	25.00
ACE inhibitors	5/24	20.83
Angiotensin AT_1_ receptor antagonist	4/24	16.66
Calcium channel blockers	4/24	16.66
Aldosterone antagonist	1/24	4.16
Metformin	6/24	25.00
Glibenclamide	4/24	16.66
Statins	3/24	12.50
NPH insulin	1/24	4.16

Values do not sum 100% because some patients were under combined pharmacological treatment. ACE: angiotensin converting enzyme.

## References

[1] Aoqui C., Chmielewski S., Scherer E., Eißler R., Sollinger D., Heid I., Braren R., Schmaderer C., Megens R. T. A., Weber C., Heemann U., Tschöp M., Baumann M. (2014). Microvascular dysfunction in the course of metabolic syndrome induced by high-fat diet. *Cardiovascular Diabetology*.

[2] Grundy S. M., Cleeman J. I., Daniels S. R., Donato K. A., Eckel R. H., Franklin B. A., Gordon D. J., Krauss R. M., Savage P. J., Smith S. C., Spertus J. A., Costa F. (2005). Diagnosis and management of the metabolic syndrome: an American Heart Association/National Heart, Lung, and Blood Institute scientific statement. *Circulation*.

[3] Alberti K. G. M. M., Eckel R. H., Grundy S. M. (2009). Harmonizing the metabolic syndrome: a joint interim statement of the international diabetes federation task force on epidemiology and prevention; National Heart, Lung, and Blood Institute; American Heart Association; World Heart Federation; International Atherosclerosis Society; and International Association for the Study of Obesity. *Circulation*.

[4] Otani H. (2011). Oxidative stress as pathogenesis of cardiovascular risk associated with metabolic syndrome. *Antioxidants and Redox Signaling*.

[5] Grattagliano I., Palmieri V. O., Portincasa P., Moschetta A., Palasciano G. (2008). Oxidative stress-induced risk factors associated with the metabolic syndrome: a unifying hypothesis. *Journal of Nutritional Biochemistry*.

[6] Sánchez-Rodríguez M. A., Martínez-Cruz M., Correa-Muñoz E., Mendoza-Núñez V. M. (2010). Relationship between metabolic syndrome components and oxidative stress in elderly community-dwelling mexicans. *Annals of Nutrition and Metabolism*.

[7] Li C. I., Kardia S. L., Liu C. S., Lin W. Y., Lin C. H., Lee Y. D., Sung F. C., Li T. C., Lin C. C. (2011). Metabolic syndrome is associated with change in subclinical arterial stiffness—a community-based Taichung Community Health Study. *BMC Public Health*.

[8] Armutcu F., Ataymen M., Atmaca H., Gurel A. (2008). Oxidative stress markers, C-reactive protein and heat shock protein 70 levels in subjects with metabolic syndrome. *Clinical Chemistry and Laboratory Medicine*.

[9] Wolin M. S. (2009). Reactive oxygen species and the control of vascular function. *American Journal of Physiology—Heart and Circulatory Physiology*.

[10] Taniyama Y., Griendling K. K. (2003). Reactive oxygen species in the vasculature: molecular and cellular mechanisms. *Hypertension*.

[11] Ceriello A., Motz E. (2004). Is oxidative stress the pathogenic mechanism underlying insulin resistance, diabetes, and cardiovascular disease? The common soil hypothesis revisited. *Arteriosclerosis, Thrombosis, and Vascular Biology*.

[12] Kleinbongard P., Dejam A., Lauer T., Jax T., Kerber S., Gharini P., Balzer J., Zotz R. B., Scharf R. E., Willers R., Schechter A. N., Feelisch M., Kelm M. (2006). Plasma nitrite concentrations reflect the degree of endothelial dysfunction in humans. *Free Radical Biology and Medicine*.

[13] Demircan N., Gürel A., Armutcu F., Ünalacak M., Aktunç E., Atmaca H. (2008). The evaluation of serum cystatin C, malondialdehyde, and total antioxidant status in patients with metabolic syndrome. *Medical Science Monitor*.

[14] Galijasevic S., Saed G. M., Diamond M. P., Abu-Soud H. M. (2003). Myeloperoxidase up-regulates the catalytic activity of inducible nitric oxide synthase by preventing nitric oxide feedback inhibition. *Proceedings of the National Academy of Sciences of the United States of America*.

[15] Davies M. J. (2011). Myeloperoxidase-derived oxidation: mechanisms of biological damage and its prevention. *Journal of Clinical Biochemistry and Nutrition*.

[16] Abu-Soud H. M., Hazen S. L. (2000). Nitric oxide modulates the catalytic activity of myeloperoxidase. *The Journal of Biological Chemistry*.

[17] Azekoshi Y., Yasu T., Watanabe S., Tagawa T., Abe S., Yamakawa K., Uehara Y., Momomura S., Urata H., Ueda S. (2010). Free fatty acid causes leukocyte activation and resultant endothelial dysfunction through enhanced angiotensin II production in mononuclear and polymorphonuclear cells. *Hypertension*.

[18] von Leitner E.-C., Klinke A., Atzler D., Slocum J. L., Lund N., Kielstein J. T., Maas R., Schmidt-Haupt R., Pekarova M., Hellwinkel O., Tsikas D., D'Alecy L. G., Lau D., Willems S., Kubala L., Ehmke H., Meinertz T., Blankenberg S., Schwedhelm E., Gadegbeku C. A., Böger R. H., Baldus S., Sydow K. (2011). Pathogenic cycle between the endogenous nitric oxide synthase inhibitor asymmetrical dimethylarginine and the leukocyte-derived hemoprotein myeloperoxidase. *Circulation*.

[19] Baldus S., Rudolph V., Roiss M., Ito W. D., Rudolph T. K., Eiserich J. P., Sydow K., Lau D., Szöcs K., Klinke A., Kubala L., Berglund L., Schrepfer S., Deuse T., Haddad M., Risius T., Klemm H., Reichenspurner H. C., Meinertz T., Heitzer T. (2006). Heparins increase endothelial nitric oxide bioavailability by liberating vessel-immobilized myeloperoxidase. *Circulation*.

[20] Nicholls S. J., Hazen S. L. (2005). Myeloperoxidase and cardiovascular disease. *Arteriosclerosis, Thrombosis, and Vascular Biology*.

[21] Cacoub P., Cambou J. P., Kownator S., Belliard J. P., Beregi J. P., Branchereau A., Carpentier P., Léger P., Luizy F., Maïza D., Mihci E., Herrmann M. A., Priollet P. (2009). Prevalence of peripheral arterial disease in high-risk patients using ankle-brachial index in general practice: a cross-sectional study. *International Journal of Clinical Practice*.

[22] Yubero-Serrano E. M., Delgado-Lista J., Peña-Orihuela P., Perez-Martinez P., Fuentes F., Marin C., Tunez I., Tinahones F. J., Perez-Jimenez F., Roche H. M., Lopez-Miranda J. (2013). Oxidative stress is associated with the number of components of metabolic syndrome: LIPGENE study. *Experimental and Molecular Medicine*.

[23] Alberti K. G. M. M., Zimmet P., Shaw J. (2005). The metabolic syndrome—a new worldwide definition. *The Lancet*.

[24] Takazawa K., Kobayashi H., Shindo N., Tanaka N., Yamashina A. (2007). Relationship between radial and central aeterial pulse wave and evaluation of central aortic pressure using the radial arterial pulse wave. *Hypertension Research*.

[25] Matthews D. R., Hosker J. P., Rudenski A. S., Naylor B. A., Treacher D. F., Turner R. C. (1985). Homeostasis model assessment: insulin resistance and *β*-cell function from fasting plasma glucose and insulin concentrations in man. *Diabetologia*.

[26] Bostom A. G., Kronenberg F., Ritz E. (2002). Predictive performance of renal function equations for patients with chronic kidney disease and normal serum creatinine levels. *Journal of the American Society of Nephrology*.

[27] Ohkawa H., Ohishi N., Yagi K. (1979). Assay for lipid peroxides in animal tissues by thiobarbituric acid reaction. *Analytical Biochemistry*.

[28] Bradford M. M. (1976). A rapid and sensitive method for the quantitation of microgram quantities of protein utilizing the principle of protein dye binding. *Analytical Biochemistry*.

[29] Xu P., Costa-Goncalves A. C., Todiras M., Rabelo L. A., Sampaio W. O., Moura M. M., Santos S. S., Luft F. C., Bader M., Gross V., Alenina N., Santos R. A. S. (2008). Endothelial dysfunction and elevated blood pressure in Mas gene-deleted mice. *Hypertension*.

[30] Misko T. P., Schilling R. J., Salvemini D., Moore W. M., Currie M. G. (1993). A fluorometric assay for the measurement of nitrite in biological samples. *Analytical Biochemistry*.

[31] Corraliza I. M., Campo M. L., Soler G., Modolell M. (1994). Determination of arginase activity in macrophages: a micromethod. *Journal of Immunological Methods*.

[32] Simão A. N. C., Dichi J. B., Barbosa D. S., Cecchini R., Dichi I. (2008). Influence of uric acid and *γ*-glutamyltransferase on total antioxidant capacity and oxidative stress in patients with metabolic syndrome. *Nutrition*.

[33] Fujita K., Nishizawa H., Funahashi T., Shimomura I., Shimabukuro M. (2006). Systemic oxidative stress is associated with visceral fat accumulation and the metabolic syndrome. *Circulation Journal*.

[34] Bandeira S. D. M., Guedes G. D. S., Fonseca L. J. S. D., Pires A. S., Gelain D. P., Moreira J. C. F., Rabelo L. A., Vasconcelos S. M. L., Goulart M. O. F. (2012). Characterization of blood oxidative stress in type 2 diabetes mellitus patients: increase in lipid peroxidation and SOD activity. *Oxidative Medicine and Cellular Longevity*.

[35] Ziobro A., Duchnowicz P., Mulik A., Koter-Michalak M., Broncel M. (2013). Oxidative damages in erythrocytes of patients with metabolic syndrome. *Molecular and Cellular Biochemistry*.

[36] Félétou M., Vanhoutte P. M. (2006). Endothelial dysfunction: a multifaceted disorder. *American Journal of Physiology: Heart and Circulatory Physiology*.

[37] Fukai T., Ushio-Fukai M. (2011). Superoxide dismutases: role in redox signaling, vascular function, and diseases. *Antioxidants & Redox Signaling*.

[38] Viroonudomphol D., Pongpaew P., Tungtrongchitr R., Phonrat B., Supawan V., Vudhivai N., Schelp F. P. (2000). Erythrocyte antioxidant enzymes and blood pressure in relation to overweight and obese Thai in Bangkok. *Southeast Asian Journal of Tropical Medicine and Public Health*.

[39] Biasucci L. M., D'Onofrio G., Liuzzo G., Zini G., Monaco C., Caligiuri G., Tommasi M., Rebuzzi A. G., Maseri A. (1996). Intracellular neutrophil myeloperoxidase is reduced in unstable angina and acute myocardial infarction, but its reduction is not related to ischemia. *Journal of the American College of Cardiology*.

[40] Kim J. A., Choi Y. S., Hong J. I., Kim S. H., Jung H. H., Kim S. M. (2006). Association of metabolic syndrome with white blood cell subtype and red blood cells. *Endocrine Journal*.

[41] Babio N., Ibarrola-Jurado N., Bulló M. (2013). White blood cell counts as risk markers of developing metabolic syndrome and its components in the PREDIMED study. *PLoS ONE*.

[42] Liu Y., Wang D., Li D., Sun R., Xia M. (2014). Associations of retinol-binding protein 4 with oxidative stress, inflammatory markers, and metabolic syndrome in a middle-aged and elderly Chinese population. *Diabetology and Metabolic Syndrome*.

[43] Laurent S., Cockcroft J., van Bortel L. (2006). Expert consensus document on arterial stiffness: methodological issues and clinical applications. *European Heart Journal*.

[44] Mitchell G. F., Hwang S.-J., Vasan R. S., Larson M. G., Pencina M. J., Hamburg N. M., Vita J. A., Levy D., Benjamin E. J. (2010). Arterial stiffness and cardiovascular events: the framingham heart study. *Circulation*.

[45] McEniery C. M., Wallace S., MacKenzie I. S., McDonnell B., Newby D. E., Cockcroft J. R., Wilkinson I. B. (2006). Endothelial function is associated with pulse pressure, pulse wave velocity, and augmentation index in healthy humans. *Hypertension*.

[46] Nelson M. R., Stepanek J., Cevette M., Covalciuc M., Hurst R. T., Tajik A. J. (2010). Noninvasive measurement of central vascular pressures with arterial tonometry: clinical revival of the pulse pressure waveform?. *Mayo Clinic Proceedings*.

[47] Kovaite M., Petrulioniene Z., Ryliskyte L., Badariene J., Dzenkeviciute V., Cypiene A., Laucevicius A., Polena S., Gintautas J. (2007). Systemic assessment of arterial wall structure and function in metabolic syndrome. *Proceedings of the Western Pharmacology Society*.

[48] Lüscher T. F., Creager M. A., Beckman J. A., Cosentino F. (2003). Diabetes and vascular disease. Pathophysiology, clinical consequences, and medical therapy: part II. *Circulation*.

[49] Wilkinson I. B., Hall I. R., MacCallum H. (2002). Pulse-wave analysis: clinical evaluation of a noninvasive, widely applicable method for assessing endothelial function. *Arteriosclerosis, Thrombosis, and Vascular Biology*.

[50] Simão A. N. C., Lozovoy M. A. B., Simão T. N. C., Venturini D., Barbosa D. S., Dichi J. B., Matsuo T., Cecchini R., Dichi I. (2011). Immunological and biochemical parameters of patients with metabolic syndrome and the participation of oxidative and nitroactive stress. *Brazilian Journal of Medical and Biological Research*.

[51] Ueyama J., Kondo T., Imai R., Kimata A., Yamamoto K., Suzuki K., Inoue T., Ito Y., Miyamoto K.-I., Hasegawa T., Hamajima N. (2008). Association of serum NO_*x*_ level with clustering of metabolic syndrome components in middle-aged and elderly general populations in Japan. *Environmental Health and Preventive Medicine*.

[52] Hornig B., Landmesser U., Kohler C., Ahlersmann D., Spiekermann S., Christoph A., Tatge H., Drexler H. (2001). Comparative effect of ACE inhibition and angiotensin II type 1 receptor antagonism on bioavailability of nitric oxide in patients with coronary artery disease: role of superoxide dismutase. *Circulation*.

[53] Grigore L., Raselli S., Garlaschelli K., Redaelli L., Norata G. D., Pirillo A., Catapano A. L. (2013). Effect of treatment with pravastatin or ezetimibe on endothelial function in patients with moderate hypercholesterolemia. *European Journal of Clinical Pharmacology*.

[54] Kashyap S. R., Lara A., Zhang R., Young M. P., DeFronzo R. A. (2008). Insulin reduces plasma arginase activity in type 2 diabetic patients. *Diabetes Care*.

[55] Katusic Z. S. (2007). Mechanisms of endothelial dysfunction induced by aging: role of arginase I. *Circulation Research*.

